# Clinical Usefulness of *Klebsiella Pneumoniae* Carbapenemase-Producing *K. Pneumoniae* Genotyping: The Experience of a Single-Center Epidemic

**DOI:** 10.20411/pai.v1i2.109

**Published:** 2017-01-09

**Authors:** Marianna Rossi, Liliane Chatenoud, Egidio Franco Viganò, Anna Maria Peri, Laura Alagna, Simone Bramati, Monica Manenti, Monica Raggi, Annalisa Cavallero, Luca Bisi, Sebastiano Leone, Guglielmo Marco Migliorino, Alessandra Bandera, Andrea Gori

**Affiliations:** 1 Division of Infectious Diseases, Department of Internal Medicine, “San Gerardo” Hospital, Monza, Italy; 2 IRCCS—Istituto di Ricerche Farmacologiche “Mario Negri,” Milan, Italy; 3 Microbiology Unit, “San Gerardo” Hospital, Monza, Italy

**Keywords:** KPC-*Kp*, carbapenemases, *Enterobacteriaceae*, genotyping profiles, drug-resistant

## Abstract

**Background:**

During the last decade, the spread of *Klebsiella pneumoniae*-carbapenemase-producing *Klebsiella pneumoniae* (KPC-*Kp*) has increased dramatically worldwide. In this scenario, growing interest has been addressed to genotyping of KPC-*Kp* strains, which emerged as an important tool for a better understanding of the epidemiological and clinical characteristics of the outbreaks.

**Methods:**

We performed a retrospective cohort study on patients infected with KPC-*Kp* during a 28-month outbreak period (January 2010–April 2012) at San Gerardo Hospital (Monza, Italy), investigating KPC-*Kp* genotypes by means of repetitive element sequence-based polymerase chain reaction (Rep-PCR).

**Results:**

We enrolled 97 patients infected with KPC-*Kp*. Rep-PCR analysis identified 5 distinct clone types, with different distribution over time. During the first 12 months of the outbreak period, only 1 clone was detected (clone A, in 47 patients), while the 4 other clones were identified over the remaining 16 months (clones C, E, and F/L in 23, 24, and 3 patients respectively). Mechanical ventilation was less frequent in patients infected with clones C/E/F/L (OR = 0.14; 95% CI: 0.05-0.37) compared to clone A, and the Charlson comorbidity index (CI) was more likely to have a score >5 in patients infected with clones C/E/F/L (OR = 7.21; 95% CI: 2.24-23.14) compared to clone A.

Overall mortality was higher in patients infected with clones C/E/F/L (13/20 patients, 65%) compared to those infected with clone A (7/20, 35%). Mortality in patients infected with clones C/E/F/L remained significantly higher even after adjusting for the potential confounding effect of comorbidities (ie, CI), with a hazard ratio (HR) of 4.65 (95% CI: 1.83-11.89).

**Conclusions:**

Our results suggested a close relationship between strain genotype and clinical outcome.

## INTRODUCTION

During the last decade, carbapenemases have been increasingly detected in *Enterobacteriaceae* [1]. Since the early 2000s, *Klebsiella pneumoniae* carbapenemase-producing *Klebsiella pneumoniae* (KPC-*Kp*) has become a significant concern, given the role of carbapenems as the last option for life-threatening infections caused by multidrug-resistant (MDR) Gram-negative bacteria [1–4]. The spread of KPC-producing strains has reached epidemic proportions in several areas, such as Southern Europe, the Northeastern USA, and the Far East [5]. The prevalence of KPCs strains varies across Europe, being higher in Greece and Italy compared to Northern Europe [6]. The EARS-Net surveillance system reported a remarkable increase in the rate of carbapenem resistance among Italian isolates, rising from 1% to 2% between 2006 and 2009, to 15% in 2010 and to 30% between 2011 and 2013 [6]. KPC-producing strains are responsible for hard-to-treat infections, mainly occurring in healthcare settings and in patients with multiple risk factors [7]. Moreover, KPC isolates are usually resistant to different classes of antibiotics, including β-lactams, fluoroquinolones, and carbapenems, representing a serious challenge for physicians. Treatment options for these MDR-isolates are often limited to colistin, gentamicin, and tigecycline, although isolates showing additional resistance to these last-resort antibiotics are increasingly detected [1].

If available, ceftazidime-avibactam is a new FDA-approved option to treat KPC-*Kp* infections, although studies about its efficacy in real-life settings are still underway [8]. Few retrospective studies have shown how combination therapy for KPC-*Kp* infections (such as tigecycline plus colistin; tigecycline plus gentamicin; tigecycline, colistin, and meropenem; tigecycline, gentamicin, and meropenem) is associated with lower mortality than monotherapy [9–10]. Nevertheless, randomized controlled trials comparing different therapeutic options for KPC-*Kp* strains are still underway, and evidence about what treatment is best for these infections has yet to emerge [1, 9, 11]. Therapeutic failures and high mortality rates, ranging from 13% to 58% in KPC-*Kp* bloodstream infections, have been reported in various studies [9–10]. Furthermore, some studies have shown how clinical outcomes can be influenced not only by antibiotic therapy but also by clonal type of strains. Gomez-Simmonds *et al*. described a borderline significant association of higher mortality rates with ST258 or isolates carrying the wzi154 allele in KPC-infected patients [12]. Moreover, some studies demonstrated that KPC ST258 strains exhibit variability of virulence-associated traits [13].

In the present study, we describe an outbreak caused by KPC-*Kp* isolates in a large Italian teaching hospital, focusing on genotyping of the strains and their association with clinical outcome.

## METHODS

This retrospective cohort study was conducted at San Gerardo Hospital, a 840-bed public teaching institution for adult and pediatric patients, with approximately 24,000 admissions/year, in Monza, Italy. The hospital has 3 intensive care units (ICUs), including a total of 25 beds, managed by separate teams of medical and nursing staff. A Solid Organ Transplant Unit is lacking. All patients diagnosed with infections due to KPC-*Kp* during the study period (January 2010 to April 2012) were included in the study. Clinical records were reviewed in order to identify demographic data, comorbidities, microbiological data, in-hospital treatment, and clinical outcomes. Comorbidities were assessed by calculation through the Charlson comorbidity index [14]. Only the first KPC-*Kp* infection episode per patient was included, while recurrent infections were excluded. The outcome measure was death within 30 days from the first positive culture. Patients enrolled in the study were only those with proven KPC-*Kp* infection, defined as the presence of a positive culture for KPC-*Kp* from either blood, bronchoalveolar lavage (BAL), urine or surgical wound swab, associated with clinical signs of systemic inflammatory response syndrome [15], in agreement with CDC criteria [16].

Antimicrobial treatment regimen was defined as adequate when it included at least 1 drug displaying *in vitro* activity against the KPC-*Kp* isolate. In non-complicated urinary catheter-related infection, catheter removal was considered to be adequate treatment.

### Microbiological study

The Vitek 2 automated system was used for isolate identification and antimicrobial susceptibility testing. Minimum inhibitory concentrations (MICs) were classified according to Clinical and Laboratory Standards Institute (CLSI) breakpoints at the time of testing (CLSI M100-S20, 2011) [17] (imipenem: susceptible, MIC < 1 mg/L, resistant, MIC > 4 mg/L; meropenem: susceptible, MIC < 1 mg/L, resistant, MIC > 4 mg/L; ertapenem: susceptible, MIC < 0.25 mg/L, resistant, MIC > 1 mg/L; gentamicin: susceptible, MIC < 4 mg/L, resistant, MIC > 16). The only exceptions were MICs for colistin and tigecycline, which were classified according to breakpoints established by the European Committee on Antimicrobial Susceptibility Testing (EUCAST version 1.3; 2011) (colistin: susceptible, MIC < 2 mg/L; resistant, MIC > 2 mg/L; tigecycline: susceptible, MIC < 1 mg/L; resistant, MIC > 2 mg/L) [18]. All isolates were screened for MBL production with use of the DPA-meropenem disk synergy test, and isolates for results that were negative were submitted to meropenem-boronic acid disk synergy test [19]. *K. pneumoniae* resistant to ≥ 1 carbapenems by susceptibility testing were considered to be KPC-*Kp*. In addition to phenotypic test, KPC presence has been confirmed by performing PCR, Xpert Carba-R test (Cepheid, Sunnyvale, CA). All strains were investigated by Rep-PCR using the DiversiLab strain typing system (Bacterial Bar-Codes; bioMérieux, Marcy l'Etoile, France) [20]. Dendrograms were generated by the DiversiLab Software. It calculated a dendogram based on the Pearson correlation coefficients. Isolates with > 95% similarity were considered to be of the same clone type.

### Statistical analysis

The overall KPC-*Kp* infection incidence rate per 10,000 days of hospitalization (KPC-IR) was estimated by merging data on KPC-*Kp* with hospital discharge files and was presented together with its 95% confidence intervals. KPC-IR was also calculated in strata of age, diagnoses at discharge, and type of ward. Considering only the 97 patients for which a KPC-*Kp* infection was proved as previously defined, all variables analyzed were described according to their distribution (number [N] and percentages [%] for each clone). Clones were named using alphabetical letters from A to L (A, C, E, F, L) according to time of appearance. Insofar as clone A was the first clone that appeared 12 months before the 4 other clones, and because it represents about 48% of the whole sample, whereas clones C, E, F, and L had far lower frequencies, in the successive analyses only 2 groups of clones were considered (ie, patients in which clone A was isolated and clones C/E/F/L). In order to identify potential associations between the clinical factors analyzed and infection with clones C/E/F/L rather than clone A, odds ratios (ORs), and their corresponding 95% confidence intervals (95% CI) were estimated for each factor. ORs were obtained applying multivariate logistic regression models, taking into account the potential confounding effect of those factors showing low collinearity and for which a *P*-value of 0.10 or lower was found in the unadjusted model (ie, age [≤ 65 or > 65]; source of KPC-*Kp* isolation [ie, bacteremia, lower respiratory tract, surgery and, considered as reference category, urinary tract infections], Charlson index [≤ 4 or > 4]; and days elapsed from hospitalization to KPC isolation [≤ 30 or > 30]). Collinearity was assessed by generating a correlation coefficient matrix. Survival distribution function within 30 days from KPC-*Kp* isolation was estimated using the Kaplan-Meier product-limit method; the nonparametric log-rank test was used to compare survival functions of patients infected with clone A compared to patients infected with clone C, E, F, or L. This analysis was also performed considering only KPC-*Kp*-related mortality within 30 days from isolation. For overall mortality, Cox proportional hazard regression analysis was performed in order to estimate hazard ratios (HR) and the corresponding 95% confidence intervals (95% CI). HR estimates were adjusted for those patient characteristics associated with the outcome with a probability of 0.10 or lower in the unadjusted model (ie, age [≤ 65 or > 65]), source of KPC-*Kp* isolation [bacteremia/other], and antibiotic therapy adequacy and KPC-*Kp* clone [A or C/E/F/L, considered as a time-dependent covariate]). The proportional hazards assumption was assessed through the creation and test of time-dependent variables in the proportional hazard model and no violation of this assumption was observed. All *P*-values presented are two-sided and a *P*-value < 0.05 indicated conventional statistical significance.

## RESULTS

**Figure 1.**
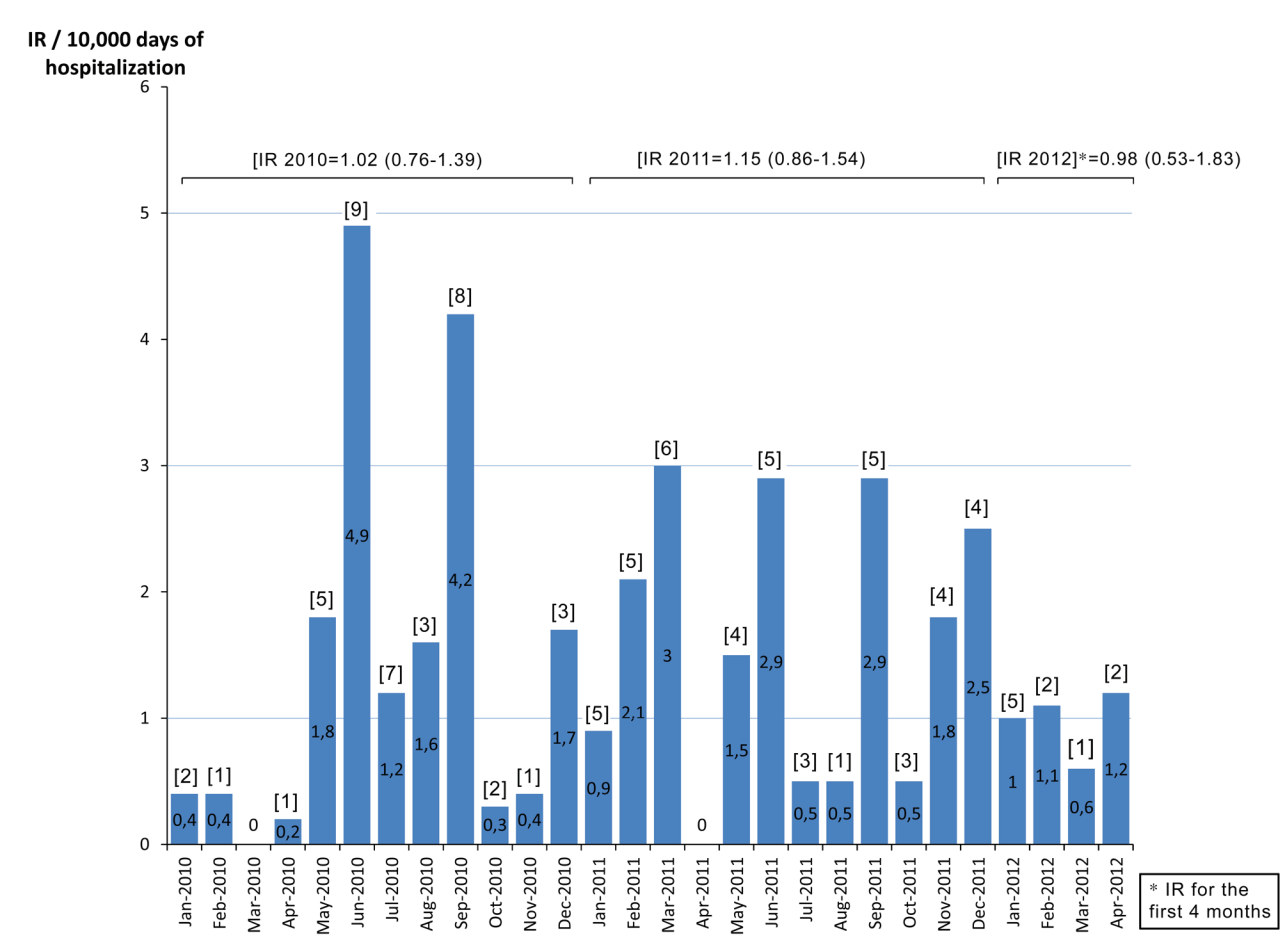
Incidence rate of KPC-*Kp* infections/10,000 days of hospitalization (IR). Number of cases is shown in brackets [n].

**Figure 2.**
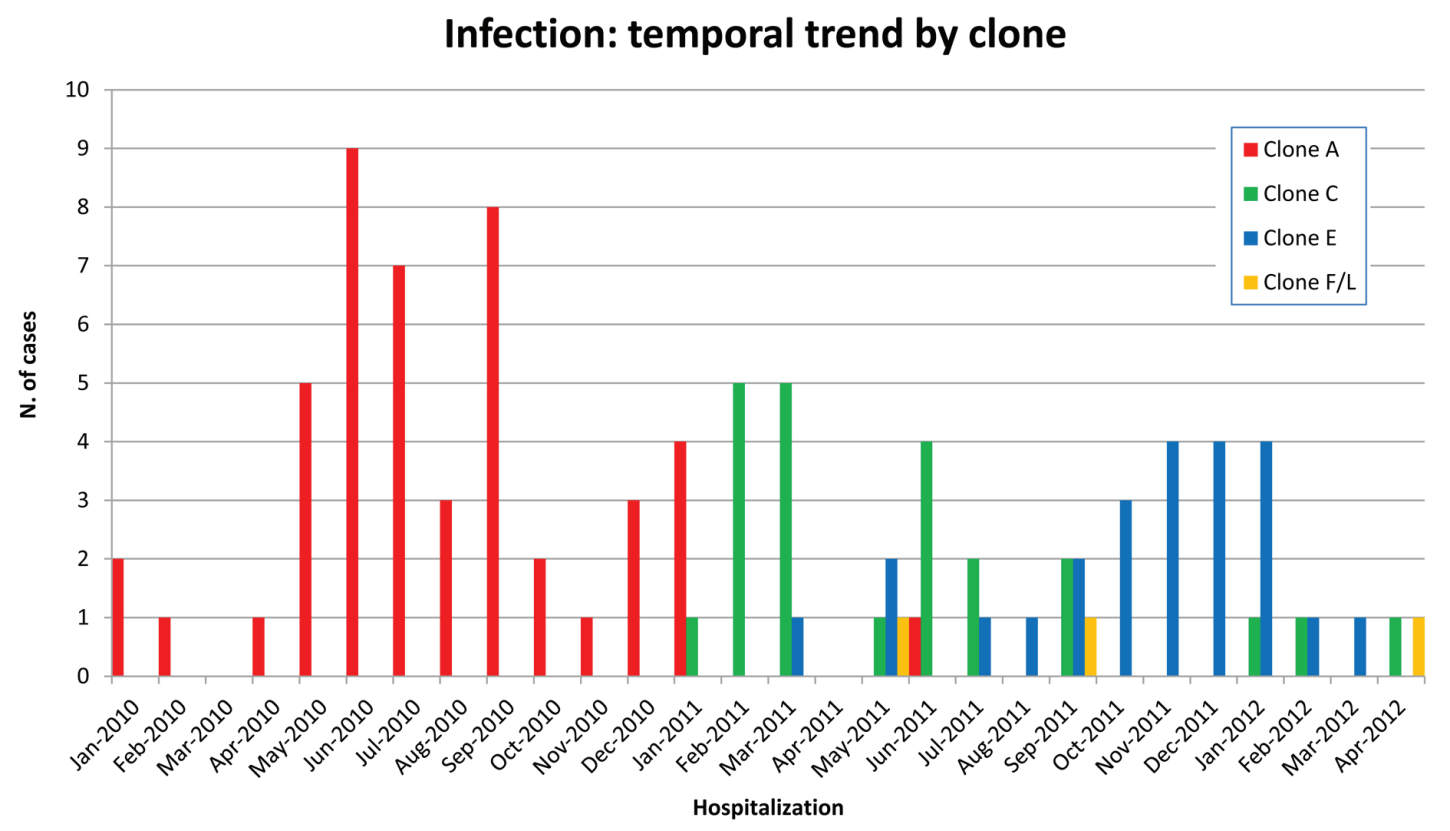
Number of KPC-*Kp* infected patients, identified by their KPC-clone, at the San Gerardo Hospital (a university public institution for adult and pediatric patients) in Monza, Italy since January 2010 (index case) and April 2012.

**Figure 3.**
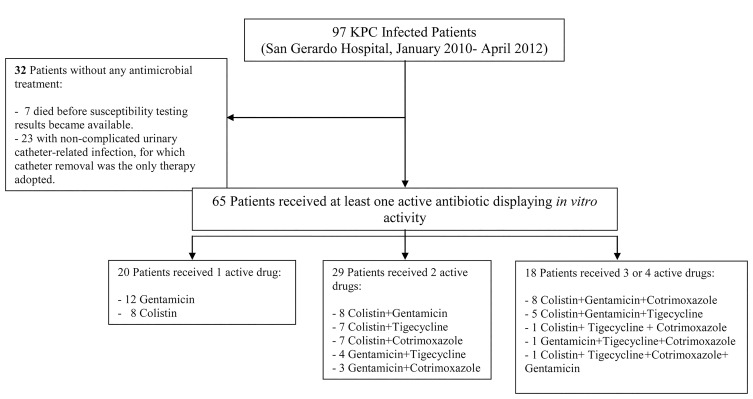
Antimicrobial treatment profile for the 97 patients considered.

Of the 85,887 patients admitted to our hospital during the study period (January 2010-April 2012), 97 developed a KPC-*Kp* infection as previously defined. In January 2010, the first KPC-*Kp* infection was diagnosed in the geriatric ward: the index case was a male patient who had been transferred from a long-term care facility. Within a few months after the index case, KPC-*Kp* infections were diagnosed in nearly every medical and surgical department of our hospital. The overall KPC-*Kp* infection incidence rate was 1.07 per 10,000 days of hospitalization (Table 1). Rates were significantly higher in older patients and in the geriatric ward and ICU compared to the surgical wards, whereas other medical wards had significantly lower IR (Table 1). KPC-*Kp* infection rate was 1.02 in 2010, 1.15 in 2011, and 0.98 in the first 4 months of 2012 (Figure 1). We performed the Cepheid Xpert Carba-R Assay PCR test, which is able to detect and differentiate the most prevalent carbapenemases gene families (KPC, NDM, VIM, IMP-1, and OXA-48), confirming the presence of KPC gene in all of the isolates. Carbapenemases other than KPC (NDM, VIM, IMP-1, and OXA-48) were not detected in any of the isolates. Rep-PCR analysis of all strains identified 5 distinct clone types that we labeled with alphabetical letters according to time of appearance: A, C, E, F, L. For the first 12 months of the KPC-*Kp* outbreak, only clone A isolates were detected (n = 39). Thereafter, clone A virtually disappeared (only 1 case was registered in June 2011), and clones C, E, F, and L were identified, although with different incidence and time windows (Figure 2). Clone C was predominant in the first 6 months of 2011, accounting for 16 of the 21 infections in that period (76%), whereas clone E was predominant in the following 6 months of the year, accounting for 75% of all of the infections of that period (n = 15) (Figure 2). The distribution of the major clinical and epidemiological characteristics is presented in Table 2—overall and stratified by clones. Clone A was the most represented clone with 47 isolates, followed by clone E (24 isolates), clone C (23 isolates), and clones F/L (3 isolates). Patients were more frequently men (66%) and about 50% of them were 66 years old or older. Comparisons between clones for each factor were performed considering clones C/E/F/L as a single group, due mainly to similarities in the distribution of the principal factors analyzed between clones C and E and to the low number of patients infected by clones F and L. At KPC-*Kp* infection diagnosis, 61 patients were hospitalized in a medical ward (23 of these in geriatric wards), 22 in a surgical ward, and 14 in ICUs, with a frequency of clones C/E/F/L compared to clone A lower in the surgical wards and ICU (OR = 0.46 and 0.20 respectively). No major differences in clone distribution emerged when patient diagnosis at discharge was considered. The Charlson comorbidity index was significantly higher in patients affected by clones C/E/F/L, with 79% and 21% of patients having a CI higher than 4 in the C/E/F/L and A clone group respectively (OR = 7.21, 95% CI: 2.24-23.14). Mechanical ventilation (presented by 36% of the total sample) was significantly less frequent in the clone C/E/F/L group; only 23% of all cases with mechanical ventilation belonged to this group. In contrast, a whopping 77% of cases with mechanical ventilation belonged to the clone A group (OR = 0.14; 95% CI: 0.05-0.37). Urine was the most common source of KPC-*Kp* isolation (46 of the 97 patients). Bacteremia was more frequent in the C/E/F/L clone group, while lower respiratory tract infection was more frequent in the clone A group. Eighty-five patients had received antibiotic therapy within 30 days before KPC-*Kp* infection, with comparable distribution (about 50% of patients), in the 2 groups of clones (ie, clone A and clones C/E/F/L). At the time of enrolment in the study, all 97 selected patients had KPC-*Kp* isolates resistant to penicillins, cephalosporins, levofloxacin, imipenem, meropenem, and amikacin. Meropenem MICs were: 1 mg/L and 2 mg/L for 5 isolates respectively; 4 mg/L for 2 isolates; 8 mg/L for 4 isolates; 16 mg/L for 19 isolates; and 32 mg/L for 62 isolates. Thirty-eight percent of isolates were resistant to cotrimoxazole. The antibiotics with major profiles of susceptibility were: colistin (89%); tigecycline (93%); and gentamicin (97%) (data not shown). Thirty-two patients received no antimicrobial treatment for KPC-*Kp* infection. Of these, 7 were already deceased by the time susceptibility testing results became available (15%, 42%, and 70% of patients infected by clone A, clone E, and clone C, respectively). Additionally, 23 patients with urinary catheter-related infection were successfully treated via catheter removal alone. Twenty patients received a single drug displaying *in vitro* activity, while 29 received two drugs, 15 received 3 drugs, whereas only 1 patient received 4 drugs (Figure 3). Among the 65 treated patients, 45 (69%) patients received an antimicrobial regimen containing colistin (in 8 [12%] patients as the only active antimicrobial) and 37 (57%) were administered other active antimicrobials (gentamicin, cotrimoxazole, tigecycline) (Figure 3). Overall, 91% of patients received an adequate antimicrobial treatment. Of the 9 patients who received inadequate antimicrobial treatment, 7 (78%) were in the C/E/F/L clones group and 2 (22%) were in the clone A group.

**Table 1.** Incidence rate of KPC-*Kp* infection/10,000 days of hospitalization (IR), overall and in strata of age, ward of isolation, and diagnoses at discharge.

	Total number of KPC-Kp infected patients		IR/10,000 days of hospitalization (95% CI)
	N	%	
**Overall (January 2010 – April 2012)**	97	(100.0)	1.07 (0.88-1.31)
**Sex**			
M	64	(66.0)	1.50 (1.17-1.92)
F	33	(34.0)	0.69 (0.49-0.97)
**Age (years)**			
19-65	26	(26.8)	0.52 (0.34-0.78)
>66	71	(73.2)	1.61 (1.28-2.02)
**Ward of Isolation**			
Geriatry	23	(23.7)	8.87 (5.90-13.4)
ICU	14	(14.4)	5.08 (3.01-8.57)
Surgery	22	(22.7)	1.89 (1.24-2.86)
Other Medical Ward	38	(39.2)	0.52 (0.38-0.71)
**Type of KPC-Kp Clone**			
Clone A	47	(48.5)	0.52 (0.39-0.69)
Clone (C/E/F/L)	40	(41.2)	0.55 (0.42-0.73)

Overall duration of hospitalization was significantly shorter for patients infected by clones C/E/F/L: 68% (n = 30) of patients with hospitalization stays shorter than 1 month were in this clone group. In contrast, percentage of hospitalization stay of 2 months or longer was 31% (n = 4). Furthermore, time elapsing from hospitalization to KPC-*Kp* isolation was shorter for patients with clones C/E/F/L. A total of 35% of patients requiring KPC-*Kp* isolation after 1 month of hospitalization belonged to this clone group, with an additional 65% in the clone A group.

**Table 2.** Distribution of major patient characteristics in strata of KPC clones with the corresponding odds ratio (OR) of being infected by clones C, E, F, or L compared to clone A.

	Total	KPC Clone						OR (95% CI)^1^	
		A		C	E	F/L	C/E/F/L		
	N	N	(%)	N			(%)		
**Sex**	64	30	(46.9)	14	17	3	(53.1)	1^2^	
M									
F	33	17	(51.5)	9	7	.	(48.5)	0.63	(0.22-1.76)
**Age (years)**	26	16	(61.5)	5	5	.	(38.5)	1^2^	
19-65									
>66	71	31	(43.7)	18	19	3	(56.3)	1.43	(0.49-4.20)
**Ward of Isolation**	23	10	(43.5)	6	6	1	(56.5)	1^2^	
Geriatry									
Other Medical Ward	38	10	(26.3)	13	13	2	(73.7)	6.13	(1.43-26.24)
Surgery	22	16	(72.7)	3	3	.	(27.3)	1.03	(0.21-5.05)
ICU	14	11	(78.6)	1	2	.	(21.4)	0.32	(0.05-2.13)
**Diagnosis at Discharge**	26	11	(42.3)	6	8	1	(57.7)	0.80	(0.28-2.31)
Infectious									
Solid and/or Hematologic Neoplasms	10	5	(50.0)	3	1	1	(50.0)	1.67	(0.31-8.91)
Diseases of the Circulatory System	21	11	(52.4)	4	5	1	(47.6)	0.87	(0. 25-2.96)
Diseases of the Respiratory System	24	11	(45.8)	6	7	.	(54.2)	1.63	(0.46-5.84)
Other Diagnosis	16	9	(56.3)	4	3	.	(43.8)	0.72	(0.19-2.68)
**Charlson Index**	68	41	(60.3)	12	14	1	(39.7)	1^2^	
0-4									
5-10	29	6	(20.7)	11	10	2	(79.3)	7.21	(2.24-23.14)
**Mechanical Ventilation**	62	20	(32.3)	20	19	3	(67.7)	1^2^	
No									
Yes	35	27	(77.1)	3	5	.	(22.9)	0.14	(0.05-0.37)
**Source of KPC Isolation**	17	6	(35.3)	5	4	2	(64.7)	1.54	(0.43-5.50)
Bacteremia									
Lower Respiratory Tract	26	18	(69.2)	4	4	.	(30.8)	0.18	(0.05-0.63)
Urinary Tract	46	19	(41.3)	12	14	1	(58.7)	1^2^	
Surgical Wound	8	4	(50.0)	2	2	.	(50.0)	0.69	(0.13-3.58)
**Antibiotic Therapy Within 30 Days Before KPC Isolation**^3^	12	4	(33.3)	5	2	1	(66.7)	1^2^	
No									
Yes	85	43	(50.6)	18	22	2	(49.4)	0.86	(0.20-3.74)
**Adequate Antimicrobial Treatment**	88	45	(51.1)	19	21	3	(48.9)	1^2^	
Yes									
No	9	2	(22.2)	4	3	.	(77.8)	6.17	(0.66-57.68)
**Total Duration of Hospitalization (days)**	44	14	(31.8)	15	14	1	(68.2)	1^2^	
1-30									
31-60	40	24	(60.0)	6	8	2	(40.0)	0.35	(0.13-1.00)
>61	13	9	(69.2)	2	2	.	(30.8)	0.28	(0.05-1.47)
**Days Elapsed From Hospitalization to KPC Isolation**	68	28	(41.2)	17	21	2	(58.8)	1^2^	
0-30 days									
>31	29	19	(65.5)	6	3	1	(34.5)	0.37	(0.13-1.10)
**Mortality Within 30 days From KPC Isolation**									
Alive	77	40	(51.9)	16	18	3	(48.1)	1^2^	
Overall Crude Mortality	20	7	(35.0)	7	6	.	(65.0)	2.88	(0.77-10.07)
KPC-attributable Mortal-ity^4^	13	2	(15.4)	6	5	.	(84.6)	11.17	(1.56-79.87)

^1^ORs adjusted, when appropriate, by age [≤ 65 or > 65], source of KPC-*Kp* isolation (ie, bacteremia, lower respiratory tract, surgery and, considered as a reference category, urinary tract infections), Charlson index [≤ 4 or > 4], and days elapsed from hospitalization to KPC isolation [≤ 30 or > 30]).

^2^Reference category.

^3^Fifty-two patients (61%), received β-lactam-β-lactamase inhibitor combinations, 50 (59%) cephalosporins, 41 (48%) fluoroquinolones, 14 (16%) carbapenems, and 13 (15%) aminoglycosides.

^4^The cause of death was septic shock in 8 patients with KPC bacteremia as well as in 1 patient with a surgical site infection and 1 patient with urosepsis. In regard to the 4 patients with KPC pulmonary infection, cause of death was respiratory distress.

**Figure 4.**
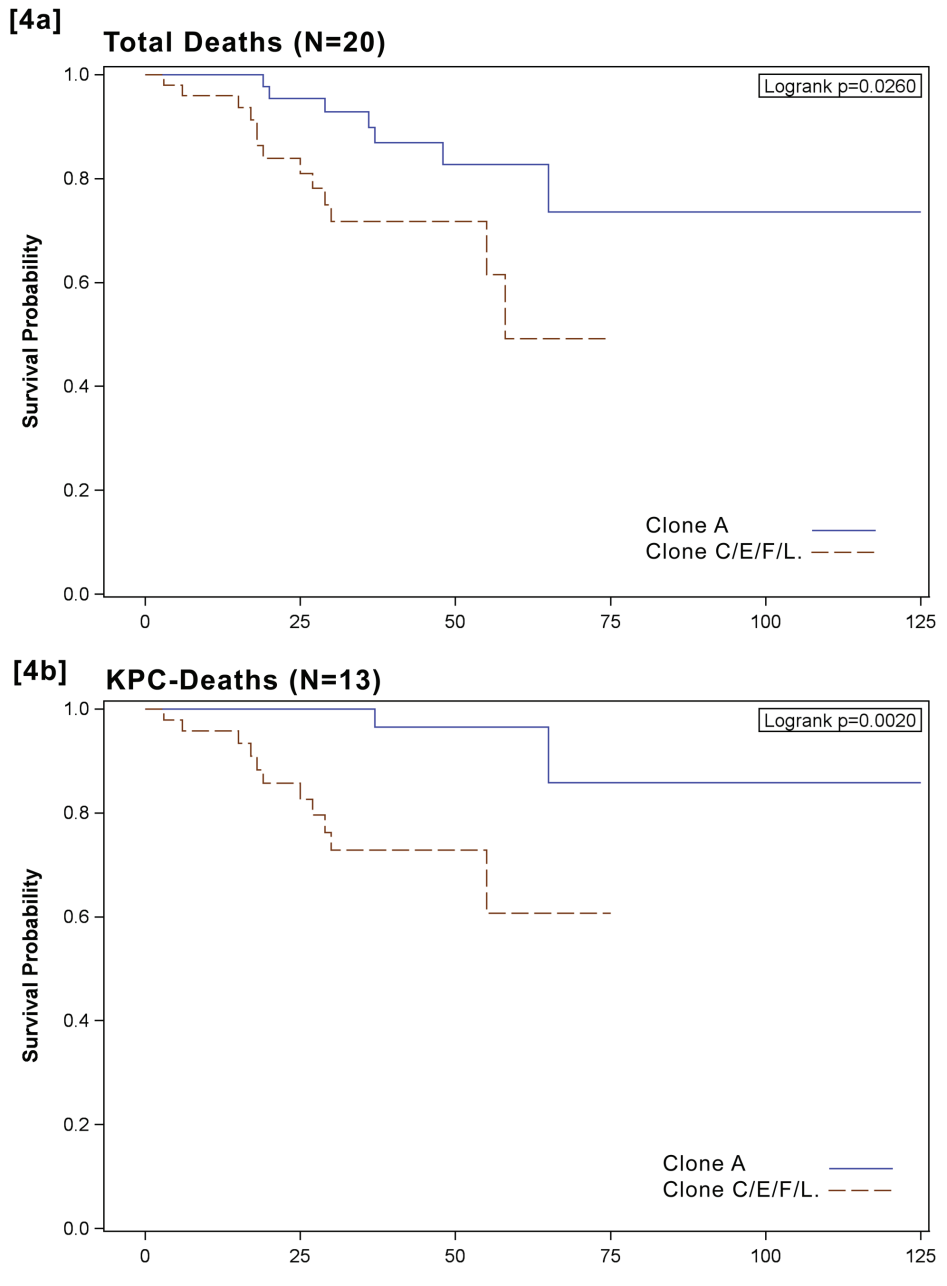
Kaplan-Meier curves showing the impact of KPC-clones (C/E/F/L: red line) versus clone A (blue line) on 30-days mortality of patients with KPC infection. Figure 2A: overall mortality, Figure 2B: KPC-related mortality

Overall mortality was higher in the C/E/F/L clone group (attributable to clones C and E only), with 65% (n = 13) of all patients dying in this group (OR = 2.88, 95% CI: 0.77-10.70), and a significant difference between clone groups was found when only KPC-*Kp*-related deaths were considered (OR = 11.17, 95% CI: 1.56-79.87). In Figure 4, Kaplan-Meier survival curves are charted for both groups of patients, those infected by clone A and those infected by clones C/E/F/L. Survival distribution was significantly lower for the group of patients infected by clones C/E/F/L (*P* = 0.05, Figure 4A), with an even greater difference being evident when only KPC-*Kp*-related mortality is considered (*P* = 0.004, Figure 4B). Among the 9 patients who did not receive adequate therapy, 7 (78%) died within 4 days of KPC-*Kp* isolation from infection-related death and 1 died after 14 days from intracerebral hemorrhage. In Table 3, rates of major risk factors were determined, factoring in overall in-hospital mortality at 30 days, as well as the respective adjusted HRs and their 95% CI. Mortality was higher in older patients (HR = 3.72, 95% CI: 0.81-17.16), in patients in ICU with respect to those in medical and surgical wards (HR=8.76, 95% CI: 2.53-30.35) and in patients receiving inadequate antibiotic treatment (HR = 8.47, 95% CI: 3.19-22.49). Mortality was also significantly higher in patients with clones C or E compared to those with clone A, even after adjusting for age class, ward where KPC-*Kp* was isolated, source of isolation, and antibiotic therapy adequacy (HR = 4.65, 95% CI: 1.83-11.83); this association persisted even when urinary tract infection were excluded (ie, 6.28 [2.24-17.59]). When KPC-*Kp*-related mortality alone was considered, adjusted HR was over 5 (HR = 6.83, 95%: 1.23-35.42); this result remained significant even when we excluded patients with inadequate antibiotic therapy (HR = 11.5 [1.20-109.0]) (data not shown).

**Table 3.** Distribution of major patient characteristics according to the outcome of death within 30 days from KPC-*Kp* isolation and the associated hazard ratio (HR) and 95% confidence intervals (CI) estimates.

	Survival Within 30 days From KPC Isolation				HR^1^ (95% CI)
	Alive (N = 77)		Dead (N = 20)		
	N	%	N	%	
**Sex**	50	(78.1)	14	(21.9)	1^2^
M					
F	27	(81.8)	6	(18.2)	1.25 (0.42-3.74)
**Age (years)**	24	(92.3)	2	(7.7)	1^2^
19-65					
> 66	53	(74.6)	18	(25.4)	3.72 (0.81-17.16)
**Ward of Isolation**	20	(87.0)	3	(13.0)	1^2^
Geriatry					
Other Medical Ward	30	(78.9)	8	(21.1)	
Surgery	19	(86.4)	3	(13.6)	
ICU	8	(57.1)	6	(42.9)	8.76 (1.53-30.35)
**Diagnosis at Discharge**	20	(76.9)	6	(23.1)	1.32(0.42-4.18)
Infectious					
Solid and/or Hematologic Neoplasms	6	(60.0)	4	(40.0)	0.63 (0.16-2.49)
Diseases of the Circulatory System	19	(90.5)	2	(9.5)	0.73 (0.16-3.38)
Diseases of the Respiratory System	19	(79.2)	5	(20.8)	0.70 (0.19-2.67)
Other Diagnosis	13	(81.3)	3	(18.8)	2.63 (0.67-10.29)
**Charlson Index**	54	(79.4)	14	(20.6)	1^2^
0-4					
5-10	23	(79.3)	6	(20.7)	0.88 (0.30-2.59)
**Mechanical Ventilation**	50	(80.6)	12	(19.4)	1^2^
No					
Yes	27	(77.1)	8	(22.9)	5.48 (1.67-17.95)
**Antibiotic Therapy Within 30 Days Before KPC Isolation**	10	(83.3)	2	(16.7)	1^2^
No					
Yes	67	(78.8)	18	(21.2)	0.98 (0.21-4.63)
**Source of KPC Isolation**	9	(52.9)	8	(47.1)	2.04 (0.78-5.37)
Bacteremia					
Lower Respiratory Tract	18	(69.2)	8	(30.8)	1.06 (0.39-2.90)
Surgical Wound	7	(93.5)	1	(6.5)	0.33 (0.09-1.29)
Urinary Tract	43	(87.5)	3	(12.5)	0.88 (0.11-7.01)
**Adequate Antimicrobial Treatment**	76	(86.4)	12	(13.6)	1^2^
Yes					
No	1	(11.1)	8	(88.9)	8.47 (3.19-22.49)
**KPC Clone**	40	(85.1)	7	(14.9)	1^2^
A					
C	16	(69.6)	7	(30.4)	
E	18	(75.0)	6	(25.0)	4.65 (1.83-11.89)
F/L	3	(100.0)	.	.	

^1^HR estimates from multivariate Cox proportional hazard model adjusted by age [≤ 65 or > 65]), source of KPC-*Kp* isolation (bacteremia/other), antibiotic therapy adequacy and KPC-*Kp* clone (A or C/E/F/L); considered as a time-dependent covariate.

^2^Reference category.

## DISCUSSION

KPC-*Kp* infections are an important public health problem and have been associated with poor patient outcomes. Moreover, the spread of KPC-*Kp* in hospitals is of serious concern, and controlling these infections poses a serious clinical challenge [4, 21–24]. In our experience, KPC-*Kp* displayed an exceptional array of multidrug resistance and remarkable efficiency of spread. After the index case, KPC-*Kp* infections were diagnosed in virtually all departments (medical, surgical wards, and ICUs) within only a few months. Moreover, during the study period (more than 2 years), the overall KPC-*Kp* rate remained stable over time. However, in analyzing the distribution of the various clones over time during the first months we observed a spread of clone A only, while in the following months new clones (C, E, F, L) were detected, with a disappearance of clone A. We observed no difference in the epidemiological characteristics of patients infected with different strains, except for the Charlson Comorbidity Index, which was higher in patients infected with clones C, E, F, or L compared to clone A. Although not statistically significant, we observed a wider diffusion in ICUs of clone A compared to other clones, likely due to a more effective control intervention on nosocomial KPC-*Kp* spread in ICUs following the first epidemic period, whereas subsequent clones were significantly more frequent in other medical wards, with traditionally lower control (mainly, general medicine wards). As expected, when analyzing 30-day mortality in KPC-*Kp*-infected patients, we observed higher mortality in patients with bacteremia. Moreover, analysis of KPC-*Kp*-related deaths showed a lower death risk in clone A infections as opposed to other clones. This observation was confirmed even after considering source of isolation as a confounding factor, suggesting a distinct role of particular strains in clinical outcomes.

An optimal treatment for KPC-*Kp* infection has not yet been established [23]. Antibiotic treatment is limited to a narrow range of choices, typically including colistin, tigecycline, and aminoglycosides. Recent findings suggest that combination treatment with colistin, tigecycline, and meropenem might improve survival rates among bacteremic patients [9, 24]. Sbrana *et al*. suggest rather that a carbapenem-sparing regimen may be advantageous in terms of decreasing selective pressure on the gut microflora of patients, and that tigecycline, combined with colistin or with gentamicin administered at high-doses, may be adequate for the treatment of KPC-*Kp* infections in selected patients [25]. Although during the study period its efficacy had not yet been assessed, it should be mentioned that a promising option for the treatment of KPC infections is currently represented by the combination ceftazidime-avibactam, as the novel β-lactamase inhibitor, avibactam, has shown to be active against different β-lactamases, including KPC. This new agent is currently approved for complicated intra-abdominal infections and complicated urinary tract infections, and a phase 3 trial confirmed its efficacy in patients infected with MDR *Enterobacteriaceae*. In Italy, ceftazidime-avibactam is currently available only for compassionate use, so that our experience with this antibiotic in clinical practice is still very limited. In our study, given the heterogeneity of treatments and the small size of our cohort, we could not analyze the effects of the different antibiotic regimens on clinical outcome and survival. However, we were able to observe a significantly lower survival time distribution for the group of patients infected by clones C/E/F/L. An even greater difference was observed when considering KPC-*Kp*-related mortality alone, at which point any delay at all in administration of antimicrobial therapy to hospitalized patients with KPC-*Kp* infection from clones C/E/F/L significantly increased mortality rates.

Some limitations of our study must be highlighted. Firstly, the relatively small sample size of this study could have affected the possibility of identifying some relevant associations as statistically significant. Nevertheless, to our knowledge, this is one of the first reports concerning an Italian setting, in which the need to associate the genotyping investigation with the clinical approach was described.

A second limitation is related to the genotyping method. Because we have not received any financial support for the study, we adopted the Rep-PCR method, in use at our center during the study period, for the typing of different clones. We were aware that other genotyping methods might obtain contrasting results; nevertheless, within study comparisons between clones it remains valid and informative. Furthermore, few studies have suggested how clones identified by rep-PCR can be mapped to specific ST types, suggesting the concept of using rep-PCR as a proxy for MLST [26–27]. On this basis, further studies based on MLST typing may be warranted to establish a correspondence between the clones we isolated and specific ST types.

In addition to these limits, our study suggested that the difference in mortality rates observed between clone A and other clones suggests that the latter group may be more virulent, although a matched case-controlled study would be required in order to evaluate the relationship between clinical outcome and distinct KPC-*Kp* clonal strains.

The different virulence among KPC clones found in the present study might be related to differences in the genes encoding for capsular polysaccharide. Molecular analysis is beyond the scope of our study but was recently described by Diago-Navarro *et al*. in 2014 [13]. The authors performed molecular capsule typing (C-patterns and wzi sequencing) of 40 carbapenem-resistant *Klebsiella pneumoniae* strains and suggested that the differences in virulence might be related to an increased production of the KPC enzyme or to the presence of a particular KPC type.

In conclusion, the differences in virulence between bacterial KPC factors remain a key point for future larger clinical prospective studies, in order to better define the importance and the exact role of clone genotyping in KPC-*Kp* infection control.

## Figures and Tables

**Supplementary Figure 1.**
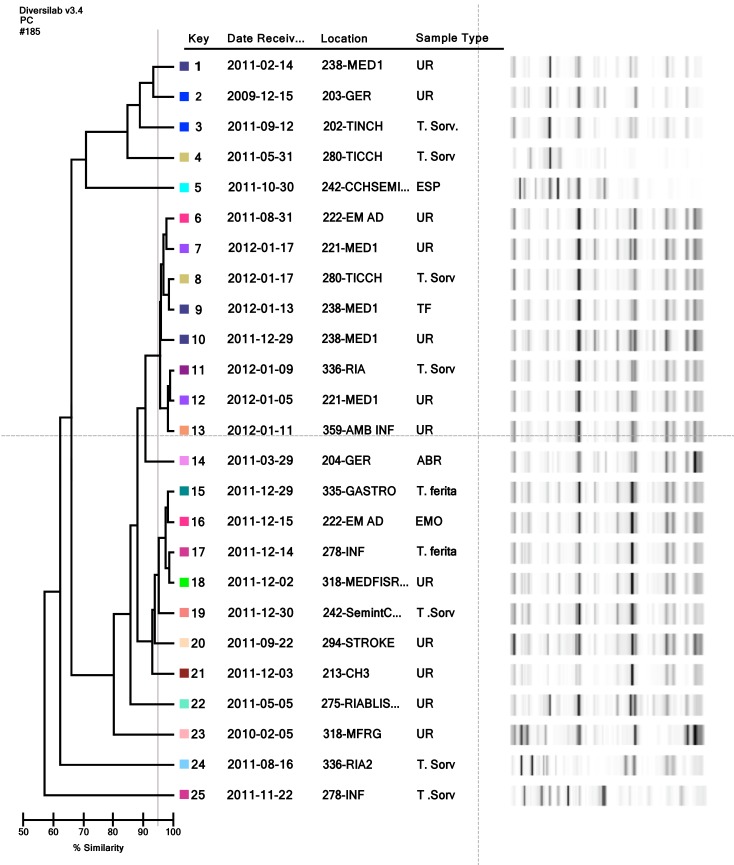
The dendogram.
